# The relationship between practice ownership and follow-up of comprehensive dental care. A Swedish register study

**DOI:** 10.2340/aos.v83.40277

**Published:** 2024-04-16

**Authors:** Bengt Franzon, Mikael Moutakis, Björn Axtelius, Sigvard Åkerman, Björn Klinge

**Affiliations:** a Department of Oral Diagnostics, Faculty of Odontology, Malmö University, Malmö, Sweden; b Department of Economics, Gothenburg University, Gothenburg, Sweden; c The Swedish Dental- and Pharmaceutical Benefits Agency, Stockholm, Sweden; d Department of Orofacial Pain and Jaw Function, Faculty of Odontology, Malmö University, Malmö, Sweden; e Division of Oral Diseases, Department of Dental Medicine, Karolinska Institutet, Stockholm, Sweden; f Department of Periodontology, Faculty of Odontology, Malmö University, Malmö, Sweden

**Keywords:** Ownership, private practice, socioeconomic factors

## Abstract

**Aims:**

The aims of this register study were:

1. To study whether the type of ownership of the dental practice was correlated with the type of dental care provided, that is public versus private ownership and professional (dentist or dental hygienist) versus non-professional ownership.
2. To study the extent of follow-up of patients who have undergone two types of treatments.

**Material and method:**

Two types of dental care were defined in the two groups studied, periodontitis/peri-implantitis and comprehensive restorative/rehabilitation. All relevant treatment codes that fall under these definitions are noted when they are performed. Also, the follow-up of each treatment code is noted. Differences in dental and socioeconomic status over time and between regions were adjusted for. A drop-out analysis was performed.

**Results:**

Dental practices owned by dentists or dental hygienists schedule follow-up appointments for patients who have undergone comprehensive restorative or rehabilitation dentistry more often than practices with other types of ownership. Dental practices owned by dentists or dental hygienists follow up patients with periodontitis and peri-implantitis less frequently.

**Conclusion:**

Type of ownership of a dental business influences the extent to which periodontal, and comprehensive restorative or rehabilitation dentistry were followed up.

## Introduction

### The focus of this study

Does the type of ownership of a dental practice affect the dental care provided? Delivery of dental care by public or privately-owned clinics has been, and remains, a major political issue in Sweden [1]. Sweden chose early on to introduce publicly owned dentistry as non-profit dental care, balancing the financial return requirements of private dentistry [2, 3]. The public dental sector, Public Dental Health Service (PDHS), today has a total share of approximately 50% of the Swedish dental care market. The private sector comprises both large and small practices [4].

The Swedish Dental Health Register, introduced in 2008, is administered by The National Board of Health and Welfare and contains data on basically all adult dental care in Sweden. The combination of a comprehensive register and diversity of ownership makes it possible to analyse whether there is a relationship between the type of practice ownership and the dental care provided [5].

### The market

The owner of a business, in this case a dental practice or clinic, is the one who ultimately determines the focus, goals, and return requirements of the business, regardless of whether it is privately or publicly owned. The option of most adults to seek dental care at a publicly or privately-owned practice or clinic is primarily a Swedish and British phenomenon [6–9].

Public dentistry in Sweden is either run under indirect political control as a limited company (PDHS) in five out of 21 Swedish regions, or as part of the administration of the region, that is under direct political control.

The first privately-owned dental care chains in Sweden were established as an effect of the market deregulation in 1999. Their market share today is approximately 5% of the private sector [4]. Most of the patients attending private dental practices are adults. Small private companies and one larger company, Praktikertjänst AB (Ptj), dominate the private dental market. Ptj is in many respects a producer cooperative owned by practicing dentists and doctors but is legally a limited company [10]. Ptj has a market share of approximately 21% of the private sector.

### Oral health and follow-up

The cost of treating oral infectious/inflammatory diseases is managed in an entirely different way from diseases in the other parts of the body [6, 11–13].

Periodontal disease and dental caries are both infectious diseases which not only have a negative impact on quality of life, but are also a great economic burden to those affected. The economic burden related to these diseases is borne by the individual, by private insurance policies, or by society. Thus, in most countries, dentistry may be regarded in economic terms as a market [11, 12].

Preventive dental care is the most cost-effective way for both individuals and society to manage the risk of caries and periodontal diseases. This is evidence-based [11, 12] and has in turn resulted in official recommendations on national public media platforms, such as those presented by WHO and EU, in national guidelines, in care programs, and clinic-related quality assurance [14–16].

Although comprehensive dental care, such as periodontal surgery, oral rehabilitation, and maintenance, is costly, it also represents an investment in long-term health, both for the individual and for society. Science, guidelines, and proven experience show the importance of follow-up (quality control), supportive treatment, treatment of illness and prevention of further illness [6, 17].

In order to investigate differences in follow-up frequency due to ownership, we created two different groups. The first group, Periodontitis and Peri-implantitis (PDPI), consisted of infection treatment. The second group, restorative or rehabilitation dentistry (REST), consisted of treatment comprising complex composite fillings, crowns, and tooth- or implant-supported bridges.

### Register study

Sweden has a unique opportunity, which many other countries lack, of retrospectively monitoring consumption of follow-up dental care [5, 18]. It is possible to determine whether there is a statistical correlation between the type of practice ownership and the care provided.

By mapping both the extent of comprehensive dental care and the extent of follow-up, that is quality control, it is also possible to study the functionality of Swedish dental care, with special reference to follow-up of comprehensive dental care.

### The aims of this register study were as follows:

1. To study whether the type of ownership of the dental practice was correlated with the type of dental care provided, that is public versus private ownership and professional (dentist or dental hygienist) versus non-professional ownership.
2. To study the extent of follow-up of patients who have undergone comprehensive dental care.

## Hypothesis

After controlling for differences in the patients’ previous dental health and socioeconomic status, there is a statistical correlation between type of ownership of the practice and recall of the patient for a follow-up appointment.

## Material and method

### Overview

The study documented follow-up (quality control) within 1 year, of patients who had undergone comprehensive dental care during the period from January 1st, 2009 to December 31st, 2017. The purpose was to determine whether follow-up was influenced by practice ownership, that is privately versus publicly owned practices and professional versus non-professional owned practices.

Follow-up is defined as a code used for dental examinations, preventive and disease-treating codes with the risk that some appointments may have been for other reasons.

### Ethical considerations

The National Board of Health and Welfare is very restrictive about disclosure of sensitive personal data linked to social security numbers. Therefore, a personal identification number key is used in communication between the authorities. The researcher obtains information with the same anonymous serial number series for both data materials [5].

A prerequisite for retrieving data on companies is that they are not disadvantaged by disclosure of confidential corporate information. Therefore, financial reporting to the dental health register was not included. Instead, production statistics were chosen to provide data. It is possible to map companies by using the organization number. This construction of small units in private dental care requires reporting at a level where individuals (dentists, dental hygienists) cannot be identified. This applies to Ptj as well.

The study was approved by The Swedish Ethical Review Authority, Dnr: 2018/112, Dnr: 2019-01537.

The Swedish Dental Health Register is a source of data for dental research and is administered by The National Board of Health and Welfare. The register, like other Swedish health registers, is based on each person’s unique social security number. This allows merging of individual patient data across other national registers. The register includes all interventions linked to diagnosis/conditions submitted to and approved by the Swedish Social Insurance Agency within the framework of state dental care, that is practically all dental care provided nationally. Data on the patients’ number of remaining and intact teeth are also included in the register. The register has been used primarily for government reports and for monitoring and developing dental care and state health care subsidies, but also for register studies [5].

The National Dental Health Register was introduced in 2008 as part of the reform of the state subsidy system for adults. During the early stages of registration procedures, some PDHS and private dental companies experienced problems in reliable delivery of data to the register. Data from 2008 were therefore excluded. The register was quality-audited in 2019 [5, 19].

The register contains four categories of information: patient data, dental care data, administrative data, and clinical data such as diagnoses, tooth number, and position, type of intervention, number of teeth, and number of intact teeth.

The dataset in this study contains all dental treatment covered by the Swedish national dental reimbursement scheme for adults, from 2009 to 2017. Additional information was sourced from the national registers from Statistikmyndigheten SCB (Statistics Sweden, SCB) and Bolagsverket (Swedish Companies Agency, SCA). SCB is responsible for official and other government statistics. SCA provides register information on Swedish companies and associations.

The Swedish dental market was defined as either PDHS, PDHS Ltd., Ptj, large dental companies/dental chains (defined as the 10 largest companies, Ptj excluded, reported to SCB December 31st, 2017) or all other private dental companies. We chose to use statistics from Privattandläkarna (Private Dentists’ Organization) based on number of patients, because it best describes the ownership when it comes to describing market shares [4]. The retrospective study used reimbursement claims data from the Swedish Dental Health Register [18]. When comparing the outcome at different clinics, differences in the characteristics of the clinics were adjusted for by logistic regression analysis. The control variables for both groups were patients’ previous dental health, age, and socioeconomic characteristics. Adjustment was also made for differences in outcome between regions and over time.

### Selection of patients

Two patient groups of adults were defined. The first group (PDPI, Periodontitis and Peri-implantitis) underwent surgery for periodontitis or peri-implantitis between 2009 and 2017. The second group, comprehensive REST, underwent treatment comprising complex composite fillings (three or more surfaces), crowns, and tooth- or implant-supported bridges during the same period. In this study, comprehensive REST were defined as involving six or more teeth.

Patients who died or emigrated within 1 year of completing REST or PDPI treatment were excluded from the analysis. Likewise, cases where there were data on the outcome but not on the control variables were excluded from the regression analysis.

### Outcome variable

Follow-up was denoted as successful if a patient was recalled for an examination, preventive care, or treatment for dental disease within 365 days of completion of the PDPI or REST treatment period. The treatment period in the Swedish subsidy system is 1 year. PDPI patients received one or more of the following treatment items, established by the Swedish dental subsidy system [19], related to periodontal surgery and dental implants: 441, 442, 443, 444 and 445. REST patients received six or more of the following treatment items: 703, 706, or 707, which comprise composite fillings involving three or more surfaces of a tooth. Items: 800, 801, 804, 805, 921 and 922: prosthetic treatment on tooth-supported constructions and 850, 852 and 853: prosthetic treatment on implant-supported constructions. One patient only appears one time in the data during the same treatment period.

The treatment codes for follow-up treatment were: Diagnostic codes (TLV series 100) 101, 102, 105, 107, 108, 111, 112, 114, 115 and 116. Prophylactic codes (TLV series 200) 201, 202, 203, 204, 205, 206, 207, 208 and 209. Disease treatment codes (The Dental and Pharmaceutical Benefits Agency (TLV) series 300) 311, 312, 321, 341, 342 and 343 [19]. Some codes have expired and others have been added due to changed regulations. The treatment period started when the first PDPI/REST treatment item was registered and ended when the last item was reported, or after 42 days whichever came first. Forty-two days was based on clinical experience.

### Control variables, statistical model, and data sources

After adjusting for background factors, logistic regression was applied to measure the statistical correlation between type of practice ownership and the outcome. The statistical correlation between clinic/practice ownership structure and the outcome was reported as an average marginal effect. The standard deviation for the average marginal effect was calculated using the delta method [20, 21].

The outcome variable was based on information as to when a patient received treatment and the treatment codes used. The Swedish National Board of Health and Welfare provided this information.

The Swedish Company Agency provided the information on the clinics’ ownership. A clinic was assumed to be owned by a dentist or a dental hygienist if it was private, but not owned by a large dental chain.

The results were adjusted for the number of active dentists per 100,000 inhabitants in the patient’s county, the patients’ dental health (the number of remaining intact and non-intact teeth registered within last 2 years before the treatment), age, and sex. The patients’ ages were coded as dummy variables for five age groups (40–49, 50–59, 60–69, 70–79, 80+). This information was provided by the Swedish National Board of Health and Welfare [5].

The results were also adjusted according to the patients’ socioeconomic characteristics (disposable household income, marital status, education, continent of birth). This information was obtained from Statistics Sweden’s longitudinal integrated database for health insurance and labor market studies (LISA) [20].

Disposable household income was defined as the patient’s total household income per year, divided by an index for household members. The index for the number of household members was adjusted to compensate for children consuming less than adults using Statistic Sweden Disposable income per consumption unit [21].

Regional differences were also adjusted for, using dummy variables for the county where the clinics were located.

### Generalizability

Although the study was based on Swedish conditions and the Swedish dental market, it should be possible to draw conclusion for other dental markets. Type of ownership of a dental business determines only partly whether the treatment provided is followed up.

### Dropout analysis

In total, 20% of observations were excluded from the statistical analysis due to missing data. The predictors with the largest share of missing data were the patient’s previous dental health (Table 1).

**Table 1:** Drop-out analysis of the observations lost in the regression analysis due to missing data on the predictor variables.

Predictor	Periodontitis/Peri-implantitis (%)	Restorative (%)
Number of intact or non-intact teeth	12	14
Income	4	3
Sex	0	0
Age	0	0
Region	3	3
Education	4	3
Marital status	0	0
Continent of birth	0	0
Number of dentists per capita	7	6
Total % of rows eliminated due to missing variables:	19	20

## Results

### Dataset

After excluding patients for whom socioeconomic and dental health data were incomplete, approximately 350,000 observations of the application of treatment codes related to PDPI or REST were available for analysis. Each observation represented the treatment of one patient.

**Graph 1:**
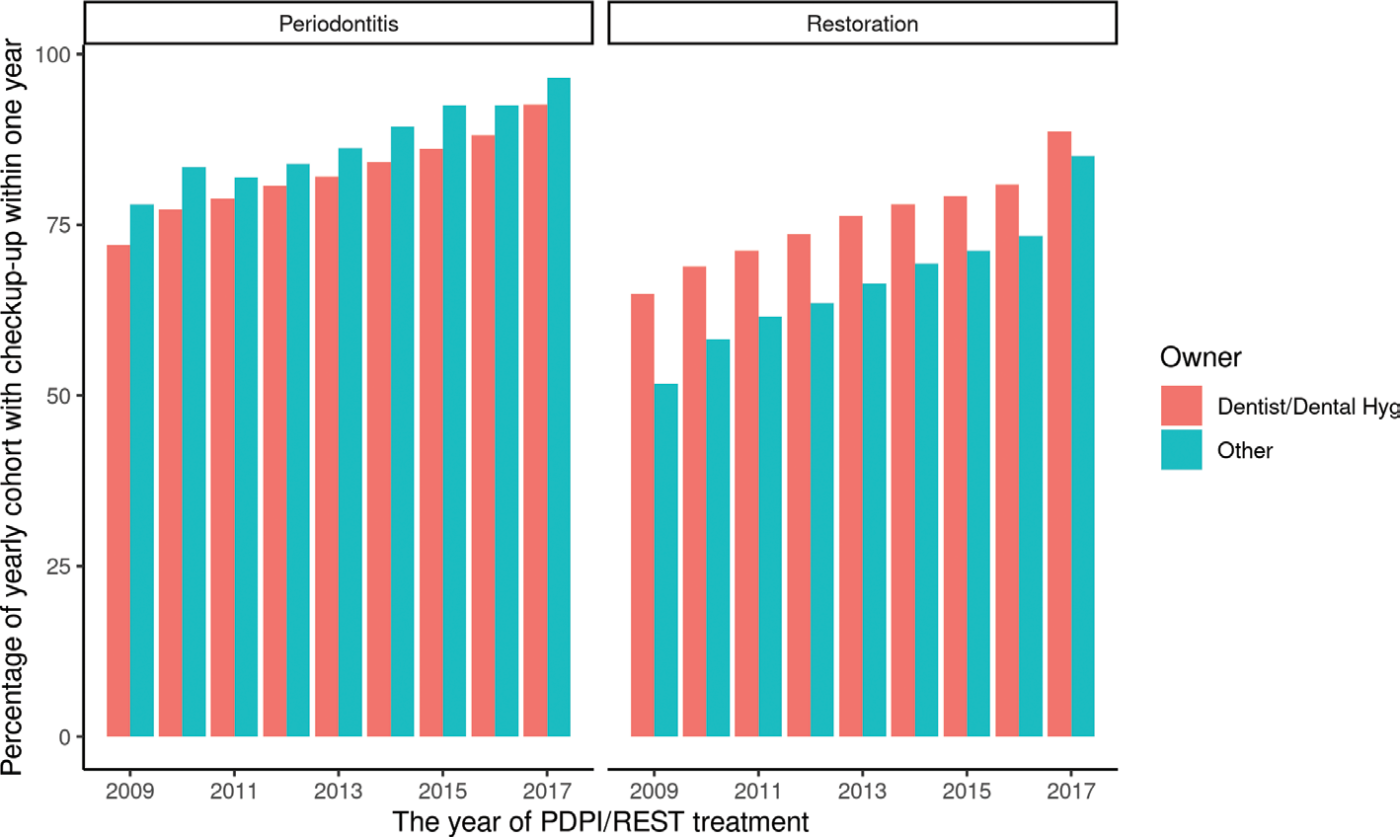
Dentist/dental hygienist owners vs other owners and the likelihood of follow-up for comprehensive periodontal treatment, and comprehensive restorative or rehabilitation dentistry, during the years 2009–2017. PDPI: comprehensive periodontal treatment, REST: comprehensive restorative or rehabilitation dentistry.

**Graph 2:**
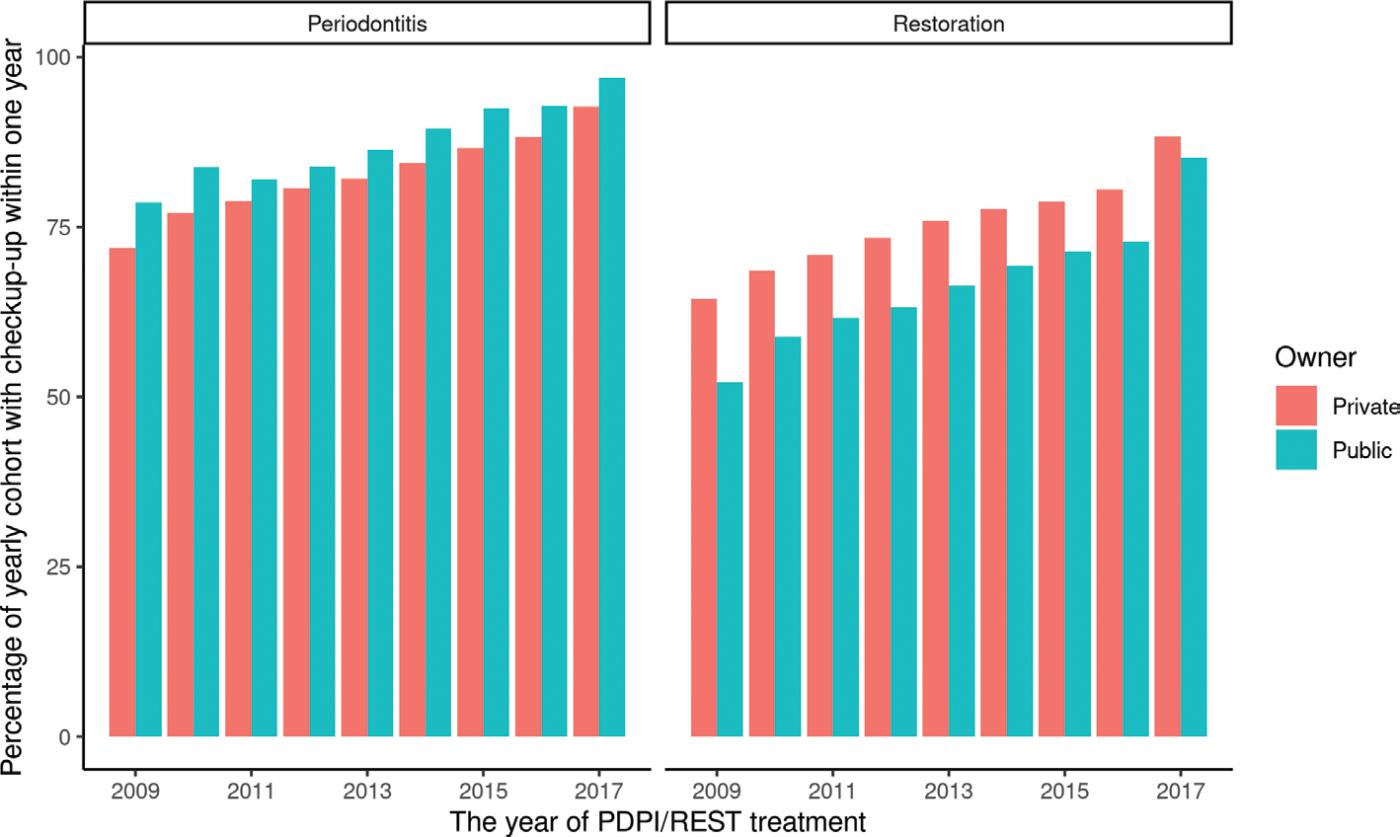
Private vs public owners and the likelihood of follow-up for comprehensive periodontal treatment, and comprehensive restorative or rehabilitation dentistry, during the years 2009–2017. PDPI: comprehensive periodontal treatment, REST: comprehensive restorative or rehabilitation dentistry.

Seventy-seven per cent of the patients were recalled for follow-up appointments within 12 months of PDPI or REST treatment. This share increased steadily over the study period. In other words, patients undergoing treatment in 2016 were much more likely to return for follow-up within 1 year than those treated in 2009 (Graphs 1 and 2).

The median patient had seven intact teeth and 15 non-intact teeth at start of treatment. The dental health data were retrieved from the last routine check-up before the periodontitis or peri-implantitis treatment. The median interval between completion of treatment and follow-up was approximately 200 days (Tables 2 and 3). The mean duration of the treatment period was 18 days.

**Table 2:** Private versus public ownership. Descriptive statistics of the observations included in the analysis, according to the type of ownership of clinic providing the treatment.

Parameter	Private	Public
Mean (SD) number of dentists per 100K inhabitants	82 (7)	81 (7)
Mean age (SD)	63 (13)	61 (13)
Mean number of items per treatment (SD)	5 (2)	4 (3)
Median (SD) number of intact teeth	7 (9)	8 (9)
Median (SD) number of non-intact teeth	15 (7)	14 (7)
Median days to monitoring	210	173
Median income per family member and month (SEK)	19,123	19,060
Number of observations	264,967	83,024
Percent female	51	51

**Table 3:** Descriptive statistics of the observations included in the analysis, according to the type of clinic providing the treatment. Clinic type is defined as either dentist/dental hygienist or non-dental owners.

Parameter	Dentist/dental hygienist	Non-dental owners
Mean (SD) number of dentists per 100K inhabitants	82 (7)	81 (7)
Mean age (SD)	63 (13)	60(13)
Mean number of items per treatment (SD)	5 (2)	4 (3)
Median (SD) number of intact teeth	7 (9)	8 (9)
Median (SD) number of non-intact teeth	15 (7)	14 (7)
Median days to monitoring	210	177
Median income per family member and month (SEK)	19,073	19,194
Number of observations	253,831	94,160
Percent female	51	51

Approximately 3/4 of the patients attended a private practice. The median income per household member was about 19,000 SEK per month. The average age was around 60 years; half the patients were female.

Eighty-three per cent of the patients received their full treatment of PDPI or REST on the same day.

### Regression analyses

After adjusting for patient characteristics, the difference in follow-up between privately and publicly owned clinics was calculated [22, 23]. We also estimated the difference between clinics owned by a dentist or dental hygienist versus all other clinics. The results were somewhat similar. Six per cent *fewer* patients treated for PDPI at a private clinic received follow-up within 12 months than patients, who had undergone similar treatment at a publicly owned clinic.

For patients who underwent REST, the results were the opposite. Nine per cent more of those treated at a private clinic (versus a public clinic) returned within 12 months for follow-up. The results were similar when clinics owned by dentists or dental hygienists were compared with all other clinics. The statistical correlations were statistically significant at the 95% level.

Of the statistically significant socioeconomic factors, age was positively correlated with the probability of follow-up within 12 months. Household income and the number of dentists per capita were positively associated with the outcome. There was a statistically significant correlation between the outcome and patients’ dental health, but only for the REST group or after combining REST and PDPI patients into one dataset. There was a positive, statistically significant correlation between income and the probability of receiving a follow-up within 12 months (Tables 4 and 5). There was a positive, statistically significant correlation between female gender and outcome for the PDPI groups, and a negative for the REST group. McFadden’s Pseudo-R2 [24, 25] was 4–6%, which in our experience is low, but not unusual for regressions at individual-level observational data (Tables 4 and 5).

**Table 4:** Predictor characteristics of background factors for dentist/dental hygienist owners versus non-dental owners and correlation characteristics with regard to the treatment groups of PDPI and REST.

Predictor	A: PDPI	B: REST	A+B
Dentist/dental hygienist owners versus non-dental owners (percentage points)	-6	9	4
Number of treatment items	+	-	-
Household income per member (SEK)	+	+	+
Female versus Male	+	-	-
Age 40–49 versus <40	+	(-)	+
Age 50–59 versus <40	+	+	+
Age 60–69 versus <40	+	+	+
Age 70–79 versus <40	+	+	+
Age 80+ versus <40	+	+	+
Number of intact teeth	(+)	+	+
Number of non-intact teeth	(-)	+	+
Number of dentists per capita	+	+	+
McFadden R2%	6	4	4
Number of observations	94,319	253,684	348,003

PDPI: comprehensive periodontal treatment, REST: comprehensive restorative or rehabilitation dentistry.

+ indicates that the correlation was positive and statistically significant (at the 95% level).

(+) indicates that the correlation was positive, but not statistically significant.

- indicates that the correlation was negative and statistically significant.

(-) indicates that the correlation was negative but not statistically significant.

**Table 5:** Predictor characteristics of background factors for private vs public owners and correlation characteristics with regard to the treatment groups of PDPI and REST.

Predictor	A: PDPI	B: REST	A+B
Private versus public owner (percentage points)	-6	9	4
Number of treatment items	+	-	-
Household income per member (SEK)	+	+	+
Female versus Male	+	-	-
Age 40–49 versus <40	+	(-)	+
Age 50–59 versus <40	+	+	+
Age 60–69 versus <40	+	+	+
Age 70–79 versus <40	+	+	+
Age 80+ versus <40	+	+	+
Number of intact teeth	(+)	+	+
Number of non-intact teeth	(-)	+	+
Number of dentists per capita	+	+	+
McFadden R2%	6	4	4
Number of observations	94,314	253,677	347,991

PDPI: comprehensive periodontal treatment, REST: comprehensive restorative or rehabilitation dentistry.

+ indicates that the correlation was positive and statistically significant (at the 95% level).

(+) indicates that the correlation was positive, but not statistically significant.

- indicates that the correlation was negative and statistically significant.

(-) indicates that the correlation was negative but not statistically significant.

## Discussion

### Register

Registers are suitable for long-term follow-up of large populations. For the present study, the Dental Health Register was chosen because it collects data on both private and public dentistry, which is necessary to ensure a complete overview of the Swedish dental market for adults.

### Follow-up

A follow-up was defined as a scheduled appointment with a dentist or a dental hygienist using examination, prevention, and disease management codes. The tariff applied to receive a state subsidy is very detailed in terms of the content of each code. It states the minimum but not the maximum content, which may result in some dentists using only code 101 (examination) and including more preventive care, that is more care is provided than is formally included. Löfgren et al described this when studying follow-up of TMJ (temporomandibular joint) disorders in a dental record study [26].

We found no scientifically established definition of comprehensive dental care. As a result, in this study, a definition was based on diagnosis and treatment codes.

We chose periodontal surgery as the definition of comprehensive periodontal treatment.

Periodontal disease and peri-implantitis are serious infectious diseases and surgery is indicated in severe cases. Comprehensive REST was defined as rehabilitation and/or reparative dentistry involving 6 or more teeth: treatment included both direct fillings and prosthetics. The rehabilitation measures included both tooth-supported and implant-supported prosthetics. We chose not to include removable dentures in rehabilitation, mainly because of the complexity of reasons for their use. Removable dentures may be both short-term and lifelong dental solutions [27].

### Ownership

Ownership of dental practices is a major issue for the dental profession. The Association of Dentists and their organizations in Europe state that dental clinics or practices should be owned and run by dentists. The guild’s analysis is that ownership by non-dentists, such as venture capitalists and others, is a risk to the quality of care [9].

In Sweden, the question of ownership of dental clinics has focused mainly on private or public ownership of dental clinics and practices. Dentistry in Sweden has a long history of both private and public ownership. The political debate on private ownership in Swedish health care is mostly about profits, ‘cream-skimming’, and unequal access to care, while debate over the issue of dental care focuses mainly on over- or undertreatment [28–31].

We chose to group Swedish dentistry based on ownership, in order to explore possible differences in follow-up after comprehensive dental care in relation to two categories:

1. Private ownership versus public ownership. PDHS and PDHS Ltd. are publicly owned, whereas Ptj, dental chains, and small dental companies are privately owned.

2. Dentist or dental hygienist-owned versus non-dentist and non-dental hygienist owned. Ptj and small dental companies are mostly owned by dentists or dental hygienists. PDHS, PDHS Ltd. and dental chains are owned by non-dentists or non-dental hygienists.

PDHS is a non-profit public-driven organization, while PHDS Ltd. is publicly owned and profit-driven [4]. Ptj is a profit-driven cooperative organization owned by dentists and medical practitioners in the company [10]. Dental chains are profit-driven, often by venture capital. Small dentist companies are profit-driven and owned by the dentist [4]. We chose to define non-dentist or non-dental hygienist ownership based on a strict interpretation of the ownership, well aware that it is an unusual way of considering dental services in Sweden, where the tradition is to compare private and public ownership. Dentists and dental organizations in Sweden tend to overlook publicly owned dentistry when problematizing the issue of ownership. Venture capital companies are regarded as the major threat to dentistry by dental organizations [9] and in Swedish public debate [30].

### Limitations of the study

One limitation of merging publicly owned PDHS and venture capital companies is that PDHS is a much larger care provider and this affects the result. PDHS has a market share almost seven times larger than all the dental care chains together (Ptj excluded) [4].

Another weakness of the study concerns dentist-owned or dental hygienist-owned-companies, where ownership of some companies may include non-dentists. For example, the shareholders of Ptj include both dentists and physicians. On the other hand, dentists have considerable influence on Ptj: since its inception, the position of chairman of the company board has been held by a dentist [10]. It would also have been of interest to the study to explore whether a non-profit dental care company stood out in comparison, but to our knowledge there are no such companies in Sweden.

There is a risk that some of the codes may have been used for other purposes than follow-up. For example, acute treatment of a completely different area of the mouth. In that respect, there is a risk that it is just a connection to dental care and not follow-up.

### Follow-up

The treatment period ranged from the first date on which the patient was charged for one of the periodontal or restorative/rehabilitation codes, up to 365 days later (Graphs 1 and 2). A recall period of 1 year is frequently applied in Swedish dental practice [6, 32]. After comprehensive dental care, three out of four patients and/or their dentist schedule a follow-up visit within a year, in order to evaluate and monitor the treatment outcome, that is the patient and the dentist consider that the treatment is so important and costly that it should be followed up. Or that the recall adds to the profit for the provider.

There are at least three possible explanations for the overall results for follow-ups. Firstly, Swedish dentistry has a tradition of preventive dentistry and regular checkups. Most dentists have recall routines for their patients. Traditionally these have been 6–12 monthly, but today more differentiated and longer intervals are applied because the dental health of the population is better and individual, risk-based assessment can be applied. In 2019, 55% of Swedes, covered by the state dental care subsidy, received dental care for some reason, and 70% within the 2-year period 2018–2019 [6].

Secondly, the Swedish national dental care subsidy system for adults is based on a treatment period of 12 months. This means that dental treatment provided during this period falls within one high-cost protection period. The high cost protection sums up all costs for 1 year and thus generates a subsidy from the subsidy system. Most PDPI and REST patients would initially have had their periodontal or prosthetic treatment reimbursed by high government subsidies. The patient’s cost for follow-up within the 12 months will be very low.

Thirdly, Swedish people have a high level of confidence in the delivery of dental services, regardless of the provider. Swedish dentistry is largely based on recall systems, whereby the care provider undertakes to keep track of the patient’s need for dental examinations [7].

### Private versus public ownership and dentist and dental hygienist versus non-dentist or non-dental hygienist ownership PDPI and REST

Tables 2 and 3 show that on average the patients at public and private clinics were similar in age, socioeconomic- and dental status. We observe the same pattern when dividing the patients into those that went to clinics owned by dentists and dental hygienists and those that didn’t.

Nor are there any differences between private and publicly owned, or dentist or dental hygienist owned and non-dentist or non-dental hygienist-owned practices or clinics (Tables 4 and 5). One reason for differentiating between dentist- and non-dentist-owned companies was to highlight the difference in the ownership structure. PDHS or PDHS Ltd. is owned by the Swedish Regions and not by dentists. PDHS and PDHS Ltd. have a much larger market share than private dental care chains, which might explain why the results for publicly owned and non-dentist-owned practices are largely the same.

We chose not to compare for-profit and non-profit companies in this study. In such a division, PDHS is measured against the private sector and PDHS Ltd.

It is unclear as to why publicly owned dental practices offer better follow-up of patients treated for periodontal disease than privately owned practices, but have a poorer record of following up repairs and rehabilitation.

PDHS and PDHS Ltd. have a market share of 26% of dental specialist treatment in Sweden. One reason is that the establishment of private specialist practices was not permitted from 1974 to 1999. This created a public monopoly of specialist care, especially outside major urban areas [2]. Today the private sector is increasing but the public sector retains a de facto monopoly in many regions. Another possible explanation is that dental hygienists are more common in public sector [4].

The combination of access to specialists in periodontology and the availability of more dental hygienists in the public sector could explain in part the difference between public and private care for the PDPI group. Treatment of periodontal disease is based on structured follow-up of health and treatment results, especially in specialist care. This and good access to dental hygienists make it possible to use the dental hygienist’s expertise as a resource.

The REST result could be explained by the structure of the private sector. Approximately 60% of adults within the state dental subsidy system are treated in the private sector [4]. Private general dental practitioners undertake most comprehensive adult dental care in Sweden. This may be one reason for the difference in REST between private and public care. Comprehensive dental care is a basic component of a private practice, as is a functioning recall system.

### Restoring health or restoring function

Regarding treatment of PDPI to restore health and REST to restore function can provide a clue to the differences in follow-up routines between private and public dentistry. Historically, people have sought dental care to relieve acute conditions [13]. The dentist ‘cured’ the patient by removing or repairing the tooth. This was sometimes followed by rehabilitation with prosthetics. This concept also characterizes some of today’s dental care [11–13]. Fees for dental care are usually based on the chairside treatment provided. That is, dental care is seen as a craft resulting in systems with a strong numerical predominance for manual therapies such as fillings, root fillings, dental implants, etc.

The organization of dental care in small units, such as private dental care, for example in Sweden, probably tends to preserve this concept of dental care, that is the clinician’s primary task is seen as the treatment of clinically manifest conditions. Public dental care in Sweden has a different background and a different focus. PDHS, just like other larger companies, is a top-down organization where the management determines the focus and organization of dental care. PDHS has, or rather has had, a general public health focus that has influenced the view of the organization [3].

### Money versus oral health

An explanation relevant to the question of why private dentists and dentist-owned companies have more focus on follow-up of REST than PDPI may be a remnant of the history of dentistry. The very close relationship between oral and systemic health is scientifically established [33, 34]. However, the focus of debate about Swedish dental care centers on economics, systems, and political distribution policy (equal dental care for all). The problem of the mechanically-oriented dentistry is highlighted in two articles published in Lancet [11, 12]. It points to the need to focus on the link between oral and systemic health and disease and on preventive dentistry.

### Socio-economics

We found a statistically significant correlation between age, sex and the probability of follow-up. (Tables 4 and 5). This is in accordance with results from other studies and government reports [6, 11, 12].

There was a statistically significant correlation between outcome and household income and the number of dentists per capita. The latest Swedish Dental Care Inquiry reviewed the dental care subsidy for adults and concluded that the subsidy has a positive effect in levelling out educational and economic inequalities. Other studies indicate that economically weaker groups have a tendency to postpone or refrain from follow-up, and instead defer seeking treatment until new dental problems arise [9, 35].

There was a positive, statistically significant correlation between the number of dentists per capita and the outcome. Globally, deterioration in dental health is often linked to a shortage of dentists.

There was a statistically significant correlation between the outcome and patients’ dental health, but only after combining PDPI and REST patients into one dataset. Although the number of teeth and the number of intact teeth is a gross estimate of dental health, it is understandable and simple. For that reason, it is also a reliable measure, which is appropriate for larger registry studies.

### Further studies

This study is explorative and other types of studies are needed to establish causal effects. Causal relationships are better established with, for example, medical record studies.

## Conclusion

Type of ownership of a dental business determines only partly whether the treatment provided is followed up.

Dental practices owned by dentists or dental hygienists scheduled follow-up appointments for patients who have undergone comprehensive REST more frequently than practices owned by non-dentists or non-dental hygienists.

Dental practices owned by dentists or dental hygienists less frequently followed-up patients with periodontal disease than practices owned by non-dentists.

## References

[1] Finansdepartementet Dir. 2015:22. Ett nytt regelverk för offentlig finansiering av privat utförda välfärdstjänster. [Government Committee Directive]. A new regulatory framework for public funding of privately performed welfare services] [Internet]. Swedish [cited 23-09-2023]. Available from: https://www.regeringen.se/contentassets/7d26d17bdd394a1c934185a445315780/ett-nytt-regelverk-for-offentlig-finansiering-av-privat-utforda-valfardstjanster-dir.-201522

[2] Franzon B, Axtelius B, Åkerman S, et al. Dental politics and subsidy systems for adults in Sweden from 1974 until 2016. BDJ Open. 2017;3:17007. 10.1038/bdjopen.2017.7 29607078 PMC5842862

[3] Lindblom C. I väntan på tandvård: Hur tandrötan blev politik [In anticipation of dentistry: How tooth decay became politic]. Dissertation. Stockholm: Carlssons Bokförlag; 2004. Swedish.

[4] Privattandläkarnas branchrapport 2020. [The association for private dental care providers in Sweden industry report 2020] [Internet]. Swedish. [cited 28-09-2023]. Available from: https://ptl.se/wp-content/uploads/2021/02/Rapport_branschrapport_2020.pdf

[5] Socialstyrelsen Tandhälsoregistret. [The National Board of Health and Welfare’s dental health register] [Internet]. Swedish [cited 15-09-2023]. Available from: https://www.socialstyrelsen.se/statistik-och-data/register/tandhalsoregistret/

[6] SOU 2021:8. [Swedish Government Official Reports 2021:8]. När behovet får styra- ett tandvårdssystem för mer jämlik tandhälsa [When the need arises – a dental care system for more equal dental health] [Internet]. [cited 23-09-2023]. Swedish. Available from: https://www.regeringen.se/rattsliga-dokument/statens-offentliga-utredningar/2021/03/sou-20218/

[7] Widström E, Augustdottir H, Byrkjeflot L, et al. Systems for provision of oral health care in the Nordic countries [Internet]. [cited 29-09-2023]. Available from: https://www.tandlaegebladet.dk/sites/default/files/articles-pdf/TB092015-702-711.pdf

[8] Sinclair E, Eaton KA, Widström E. The healthcare systems and provision of oral healthcare in European Union member states. Part 10: comparison of systems and with the United Kingdom. Br Dent J. 2019;227(4):305–10. 10.1038/s41415-019-0661-4 31444448

[9] Corporate Dentistry in Europe CED Resolution, CED-DOC-2019-045-FIN-E. [cited 10-01-2019] Available from: https://www.omd.pt/content/uploads/2019/01/ced-corporate-dentistry-en-2018.pdf

[10] Praktikertjänst tandvård [Internet]. [cited 29-09-2023]. Swedish. Available from: https://www.praktikertjanst.se/tandvard/

[11] Peres MA, Macpherson LMD, Weyant RJ, et al. Oral diseases: a global public health challenge. Lancet. 2019;394(10194):249–60. 10.1016/S0140-6736(19)31146-8. Erratum in: Lancet. 2019 Sep 21;394(10203):1010. PMID: 31327369. 31327369

[12] Watt RG, Daly B, Allison P, et al. Ending the neglect of global oral health: time for radical action. Lancet. 2019;394(10194):261–72. 10.1016/S0140-6736(19)31133-X 31327370

[13] Otto M. Teeth: the story of beauty, inequality, and the struggle for oral health in America. New York: New Press; 2016.

[14] WHO health topics oral health. [cited 14-06-2021]. Available from: https://www.who.int/health-topics/oral-health/#tab=tab_1

[15] Hugoson A, Koch G, Johansson S. Konsensuskonferens oral hälsa. [Consensus conference Oral Health]. Jönköping: Förlagshuset Gothia; 2003. Swedish.

[16] Socialstyrelsen. [The National Board of Health and Welfare] [Internet]. [cited 23-09-2023]. Swedish. Available from: https://www.socialstyrelsen.se/kunskapsstod-och-regler/regler-och-riktlinjer/nationella-riktlinjer/riktlinjer-och-utvarderingar/tandvard/

[17] Sandberg H, Fors U. Den nivågrupperade modellen – riskgruppering för bättre tandhälsa. [The level-grouped model – risk grouping for better dental health]. Swed Dent J. 2007;31:171–179. Swedish. 18220220

[18] Ljung R, Lundgren F, Appelquist M, et al. The Swedish dental health register – validation study of remaining and intact teeth. BMC Oral Health. 2019;19(1):116. 10.1186/s12903-019-0804-7 31208416 PMC6580593

[19] TLV [The Dental and Pharmaceutical Benefits Agency] [Internet]. [cited 29-09-2023]. Available from: https://www.tlv.se/tandvard/regelverk-om-tandvardsstodet.html. Swedish.

[20] SCB. Longitudinell integrationsdatabas för sjukförsäkrings-och arbetsmarknadsstudier (LISA) [Statistics Sweden. Base of longitudinal integration dates for health and labor market health insurance (LISA)] [Internet]. [cited 29-09-2023]. Swedish. Available from: https://www.scb.se/LISA.

[21] SCB. Disponibel inkomst per konsumtionsenhet för hushåll 20-64 år efter hushållstyp 2019]. [Statistics Sweden. Disposable income per consumption unit for households 20–64 years by household type 2019] [Internet]. [cited 29-09-2023]. Swedish. Available from: https://www.scb.se/hitta-statistik/temaomraden/jamstalldhet/ekonomisk-jamstalldhet/inkomster-och-loner/.

[22] Greene WH. Incidental truncation and sample selection. Econometric analysis. 7th ed. Boston, MA: Pearson; 2012.

[23] Leeper TJ. Interpreting regression results using average marginal effects with R´s margins. [cited 12-12-2023]. Available from: https://cran.hafro.is/web/packages/margins/vignettes/TechnicalDetails.pdf

[25] McFadden D. Regression-based specification tests for the multinomial logit model. J Econometr. 1987;34(1–2):63–82. 10.1016/0304-4076(87)90067-4

[26] McFadden D. Conditional logit analysis of qualitative choice behavior. Frontiers in econometrics. New York, NY: Academic Press; 1974, pp. 105–142.

[27] Löfgren A. Recognition of temporomandibular disorders: validity and outcome of three screening questions. Dissertation. Umeå: Umeå University, Odontological dissertations; 2017, p. 137.

[28] Wöstmann B, Budtz-Jørgensen E, Jepson N, et al. Indications for removable partial dentures: a literature review. Int J Prosthodont. 2005;18(2):139–45. 15889662

[29] SOU 2007:19 Friskare tänder till rimliga kostnader [Swedish Government Official Reports: Healthier teeth – at a reasonable cost] [Internet]. [cited 12-12-2023]. Swedish. Available from: https://www.regeringen.se/rattsliga-dokument/statens-offentliga-utredningar/2007/03/sou-200719/.

[30] Cheng TC, Haisken-DeNew JP, Yong J. Cream skimming and hospital transfers in a mixed public-private system. Soc Sci Med. 2015;132:156–64. 10.1016/j.socscimed.2015.03.035 25813730

[31] The social democratic labor party in Sweden [Internet]. [cited 29-09-2023]. Swedish. Available from: https://www.socialdemokraterna.se/var-politik/a-till-o/vinster-i-valfarden.

[32] Sievers J. Hon vill se smarta flöden. [She wants to see smart flows] [Internet]. Tandläkartidningen [Internet]. 2019 [cited 23-09-2023];7:28–32. Swedish. Available from: https://www.tandlakartidningen.se/nyhet/hon-vill-se-smarta-floden/

[33] SKAPA, Svenskt kvalitetsregister för karies och parodontit. Årsrapport 2019. [Swedish Quality Register for Caries and Periodontitis. Annual report 2019]. [cited 2023-09-29]. Swedish.

[34] Genco RJ, Sanz M. Clinical and public health implications of periodontal and systemic diseases: an overview. Periodontol 2000. 20620;83(1):7–13. 10.1111/prd.12344 32385880

[35] Holmstrup P, Damgaard C, Olsen I, et al. Comorbidity of periodontal disease: two sides of the same coin? An introduction for the clinician. J Oral Microbiol. 2017;9(1):1332710. 10.1080/20002297.2017.1332710 28748036 PMC5508374

[36] Folkhälsomyndigheten [Public Health Agency of Sweden] [Internet]. [cited 04-09-2023]. Swedish. Available from: https://www.folkhalsomyndigheten.se/publikationer-och-material/publikationsarkiv/j/jamlik-tandhalsa/?pub=59629.

